# Metagenomic and metabolomic analyses reveal synergistic effects of fecal microbiota transplantation and anti-PD-1 therapy on treating colorectal cancer

**DOI:** 10.3389/fimmu.2022.874922

**Published:** 2022-07-15

**Authors:** Jiayuan Huang, Xing Zheng, Wanying Kang, Huaijie Hao, Yudan Mao, Hua Zhang, Yuan Chen, Yan Tan, Yulong He, Wenjing Zhao, Yiming Yin

**Affiliations:** ^1^ School of Medicine, Shenzhen Campus of Sun Yat-Sen University, Shenzhen, China; ^2^ Department of Research and Development, Shenzhen Xbiome Biotech Co. Ltd., Shenzhen, China; ^3^ Guangdong Provincial Key Laboratory of Digestive Cancer Research, Digestive Diseases Center, The Seventh Affiliated Hospital, Sun Yat-Sen University, Shenzhen, China

**Keywords:** fecal microbiota transplantation, anti-PD-1 therapy, immunotherapy, colorectal cancer, *Bacteroides*

## Abstract

Anti-PD-1 immunotherapy has saved numerous lives of cancer patients; however, it only exerts efficacy in 10-15% of patients with colorectal cancer. Fecal microbiota transplantation (FMT) is a potential approach to improving the efficacy of anti-PD-1 therapy, whereas the detailed mechanisms and the applicability of this combination therapy remain unclear. In this study, we evaluated the synergistic effect of FMT with anti-PD-1 in curing colorectal tumor-bearing mice using a multi-omics approach. Mice treated with the combination therapy showed superior survival rate and tumor control, compared to the mice received anti-PD-1 therapy or FMT alone. Metagenomic analysis showed that composition of gut microbiota in tumor-bearing mice treated with anti-PD-1 therapy was remarkably altered through receiving FMT. Particularly, *Bacteroides* genus, including FMT-increased *B. thetaiotaomicron*, *B. fragilis*, and FMT-decreased *B. ovatus* might contribute to the enhanced efficacy of anti-PD-1 therapy. Furthermore, metabolomic analysis upon mouse plasma revealed several potential metabolites that upregulated after FMT, including punicic acid and aspirin, might promote the response to anti-PD-1 therapy *via* their immunomodulatory functions. This work broadens our understanding of the mechanism by which FMT improves the efficacy of anti-PD-1 therapy, which may contribute to the development of novel microbiota-based anti-cancer therapies.

## Introduction

The application of immune checkpoint inhibitors (ICIs) has led to remarkable advances in the treatment of a wide range of cancers, including melanoma, non-small-cell lung cancer (NSCLC), gastric cancer, and breast cancer (1). Antibodies targeting the programmed cell death protein 1 (PD-1) are the most widely used ICIs, which work by blocking the binding between PD-1 receptor of T cells and PD-L1 ligand of tumor cells, and restoring the function of T cells that recognizes and eliminates tumor cells (2). ICI therapy has saved numerous lives since its approval in 2014 and could maintain long-term disease control in ICI responders. However, in terms of curing colorectal cancer (CRC), the majority of patients would present non-response to anti-PD-1 treatment due to the insufficient tumor-infiltrating lymphocytes (TILs) in the tumor microenvironment (TME) (3, 4). Only approximately 10% of patients with CRC, which are mismatch repair deficient (dMMR) or microsatellite instability high (MSI-H) subtypes, could benefit from anti-PD-1 therapy (5, 6). Therefore, it is important to develop novel strategies to optimize our current ICI therapy.

Human intestine harbors more than 10^13^ microorganisms, which play a key role in mediating human health and disease *via* shaping systemic and local immune functions (7). Since 2015, multiple studies have elucidated that the composition of gut microbiota was associated with the efficacy of anti-PD-1 therapy (8, 9). Notably, three groups (10–12) reported their work in 2018 observing highly diversified bacterial features (i.e. high abundance of *Akkermansia, Ruminococcus*, and *Bifidobacterium*) were individually related to the favorable clinical outcomes. The mechanisms by which gut microbiota improves anti-PD-1 efficacy involve the increased abundance of beneficial bacteria, enhancement of dendritic cell (DC) maturation, increased activity of anti-tumor CD8^+^ T cells, and the promotion of T cell tumor infiltration (13). These findings suggest the potential approach to enhancing the effect of immunotherapy *via* regulating gut microbes (14).

Fecal microbiota transplantation (FMT) is a biomedical technology of transplanting functional microbiota into patients, to cure diseases *via* restoration of gut microbiota with normal composition and functions (12). FMT has been employed clinically as a main or adjunctive approach in treating a number of diseases, including *Clostridium difficile* infection, inflammatory bowel diseases, and irritable bowel syndrome (15). In 2021, two independent clinical studies demonstrated that FMT could promote the efficacy of anti-PD-1 therapy in 3/10 and 6/15 patients with PD-1-refractory melanoma, respectively (16, 17). Genes associated with peptides presentation by antigen-presenting cells (APCs) through MHC class I and IL-1 mediated signal transduction were upregulated in melanoma patients after FMT treatment (16). Another study demonstrated that patients with epithelial tumors who responded to the combinational treatment of FMT and ICI exerted increased compositions of CD8^+^ T cells, T helper 1 (Th1) cells, and APCs in the tumor microenvironment, while a reduction of myeloid-derived suppressor cells infiltration was observed (10). Animal experiments elucidated that fecal transplantation into mouse models for lung cancer led to superior tumor suppression (18). However, the detailed mechanism and the applicability of this combination therapy in other cancer types require to be further illustrated.

In this study, we evaluated the antitumor efficacy of FMT from healthy human in combination with anti-PD-1 immunotherapy using CRC tumor-bearing mouse models and investigated the underlying mechanisms through multi-omics approaches. Our results provide a potential mechanistic basis of the synergistic effects of FMT and anti-PD-1 therapy on treating colorectal cancer, which will expand our knowledge on the mechanism of immunotherapy and assist with the development of novel anticancer therapy through modulating microbiota.

## Methods

### Animals

All animal experiments were conducted at Crown Biosciences Co. Ltd. (Taicang, China) and approved by its Institutional Animal Care and Use Committee (approval number: E4756-B1901). Female BALB/c mice were purchased from Shanghai Lingchang Biological Technology Co. Ltd. (animal certificate number: 20180003003129). All mice were housed under specific-pathogen-free conditions with ingested pellet food (radio-sterilized with cobalt 60) and autoclaved water provided ad libitum.

### FMT production

Stool samples from healthy human donors with informed consent (volunteer number: 20190382) were collected using sterile boxes and processed within 2 h, as previously described (19). In a sterile anaerobic environment, the samples were thoroughly mixed with sterile normal saline (mass: volume = 1:5). Subsequently, filter bags with apertures of 1 mm, 0.25 mm, and 0.05 mm were used to remove solid particles and impurities in the stool samples. The filtered liquid was centrifuged at 5500 g at 4°C for 5 min, and the precipitation was collected. Bacterial viable counting was conducted *via* flow cytometry and anaerobic plate counting. The bacterial solution was adjusted to 0.83×10^11^ colony forming units per mL (CFU/mL), and mixed with autoclaved glycerol, frozen at −80°C until next use.

### Cell culture

CT26 mouse colon carcinoma cells (one of the most commonly used murine tumor models) were obtained from the Shanghai Institute of Life Sciences (CAT#: TCM37). Cells were cultured in RPMI 1640 culture medium (Gibco) supplemented with 10% fetal bovine serum (FBS) (Excell) and were cultured in a humidified incubator at 37°C, 5% CO_2_. CT26 cells at the exponential growth stage were suspended in PBS for subcutaneous tumor inoculation in mice.

### Tumor-bearing mouse model

**Figure 1:**
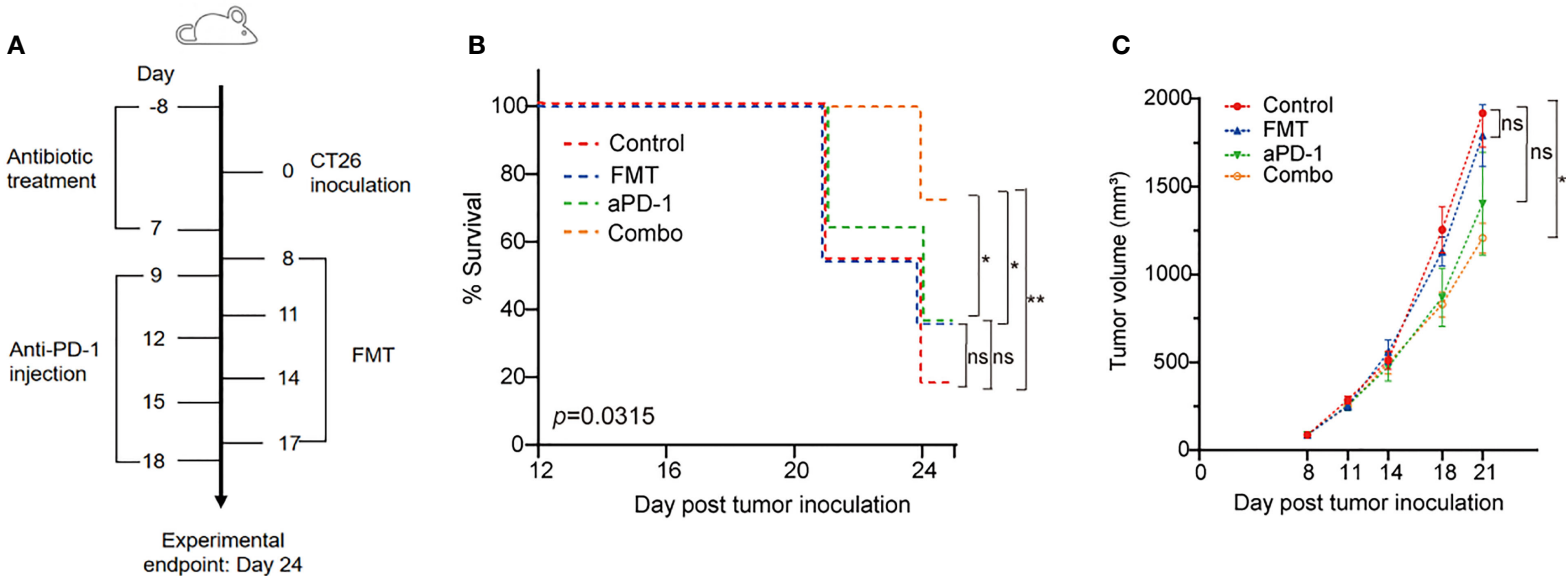
FMT and PD-1 antibody exerted synergistic anti-tumor effect in the CT26 tumor-bearing mice. **(A)** Schematic diagram of this study. **(B)** Survival curve of the CT26 tumor-bearing mice treated with FMT, aPD-1 or the combination. Statistical differences among four groups were examined using log-rank (Mantel-Cox) tests. *Post hoc* pair-wise comparisons were performed; *, *p*-value < 0.05; **, *p*-value < 0.01. **(C)** Tumor growth curves of the CT26 tumor-bearing mice treated with FMT, aPD-1 or the combination. Data are represented as mean ± SD (n = 10). Statistical differences were examined using Dunnett’s test; *, *p*-value < 0.05.

Mice (7-8 weeks old) were inoculated with 5×10^5^ CT26 cells per mouse by subcutaneous injection at Day 0 (**Figure 1A**). A total of 40 mice were randomly divided into four groups: Saline plus Rat IgG2a (designated as Control), FMT plus Rat IgG2a (FMT), Saline plus PD-1 antibody (aPD-1), and FMT in combination with PD-1 antibody (Combo). Sterile normal saline (200 μL per dose) or FMT (5×10^9^ CFU/mouse) was administered by oral gavage on Days 9, 12, 15, and 18; Rat IgG2a (200 μg/mouse, Lenico) and PD-1 antibody (200 μg/mouse, RMP1-14, Lenico) was given by intraperitoneal injection on Days 8, 11, 14, and 17. On Day 24, the endpoint of the experiment, feces, blood, and tumors of tumor-bearing mice were collected, and tumor volume was determined as length × width^2^ × 0.5. Survival rate was defined as the percentage of mice with a tumor volume of less than 2,000 mm^3^ in each group.

### Antibiotic treatment

From eight days before the tumor inoculation (Day -8) to Day -4, antibiotics were added to the drinking water in proportion, including ampicillin 1 (mg/mL), neomycin (1 mg/mL), metronidazole (1 mg/mL), vancomycin (0.5 mg/mL). From Day -3 to Day 7, ampicillin 1 mg/mL was added to the drinking water, and the mixture of metronidazole 10 mg/mL, neomycin 10 mg/mL, vancomycin 5 mg/mL, and amphotericin B 0.1 mg/mL was orally gavaged into each mouse twice a day, 200 μL each time.

### Fecal DNA extraction and metagenomic analysis

Total genomic DNA of mouse fecal samples was extracted using QIAamp PowerFecal Pro DNA Kit (Qiagen, CAT#: 51804), according to the manufacturer’s instructions. The concentration was measured by Qubit and the integrity of DNA bands was detected by agarose gel electrophoresis. Library construction and sequencing (Illumina NovaSeq 6000 platform) were performed at Novogene. Following data analyses were performed using KneadData, MetaPhlAn 2.0 and HUMAnN 2.0 with default settings (20).

### Untargeted metabolomic analysis

Mice blood samples were mixed with ice-cold methanol (3:1, v:v), and centrifuged with 12,000 rpm at 4°C for 10 min. The supernatant was collected and centrifuged at 12,000 pm at 4°C for 5 min. The sample extractions were analyzed using an LC-ESI-MS/MS system (UPLC, Shim-pack UFLC Shimadzu CBM A system; MS, QTRAP^®^ system). Chromatographic separation was carried out on a Waters ACQUITY UPLC HSS T3 C18 (1.8 µm, 2.1 mm*100 mm) column. Subsequently, the mass spectrometry separation was carried out using electrospray ionization (ESI) in the positive and negative mode (21). Following untargeted metabolomic data analysis was performed using MetaboAnalyst 4.0 with default settings (22).

### Statistical analysis

Statistical analyses were performed using R programming (version 4.0.3) and GraphPad Prism (version 8.0.2). Linear discriminant analysis effect size (LEfSe) was applied to identify differential species based on relative abundance using the Galaxy platform (http://huttenhower.sph.harvard.edu/galaxy). One-way analysis of variance (ANOVA) was performed to illustrate differential bacterial species and blood metabolites among multiple groups. False positive rate (FDR) method was employed to adjust the *p*-values when multiple comparisons were undertaken. Spearman’s correlation analysis was used to illustrate the relationship between bacterial species and metabolites.

## Results

### FMT improved the efficacy of aPD-1 in tumor-bearing mouse model

We evaluated tumor volume and survival rate in CT26 tumor-bearing mice treated with FMT or aPD-1 either alone or in combination (**Figure 1A**). The Combo group showed the highest animal survival rate (70% vs. 10%, 30%, and 30% in control, FMT, and aPD-1 groups, respectively) on Day 24 after tumor incubation (**Figure 1B**). Log-rank (Mantel-Cox) tests showed a superior survival rate of mice treated with the combination compared to those treated with FMT or aPD-1 alone (**Figure 1B**). Consistently, compared with the Control group (tumor volume 1916.9 ± 193.0 on Day 21), the Combo group exhibited a significant tumor suppression (tumor volume 1206. 6 ± 86.4, *p*-value = 0.045), while the FMT and aPD-1 groups showed the tumor volumes of 1790.4 ± 176.3 (*p*-value = 0.945) and 1402.6 ± 293.2 (*p*-value = 0.188), respectively (**Figure 1C**). These results showed that the combinational therapy had a superior effect than either monotherapy alone in treating CT26-bearing mice in terms of both survival rate and tumor control.

### FMT altered the composition of gut microbiota in tumor-bearing mice treated with aPD-1.

**Figure 2:**
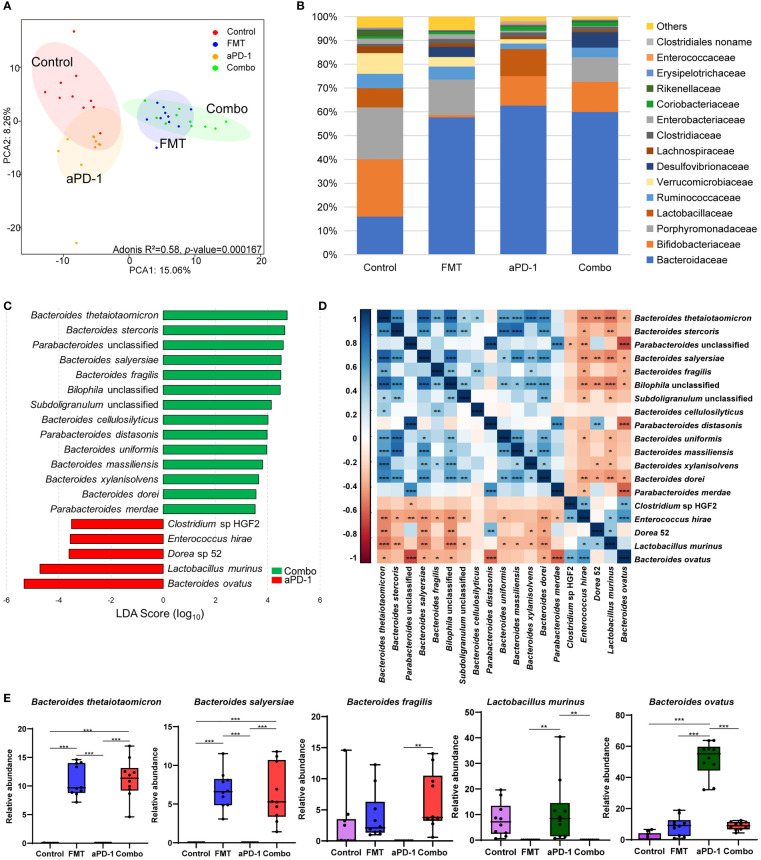
FMT altered the composition of gut microbiota in CT-26 tumor-bearing mice receiving anti-PD-1 therapy. **(A)** Principal components analysis (PCA) plot of the gut microbiota from mice. **(B)** Relative abundance of top 15 bacterial families in different groups. **(C)** LEfSe analysis showing differentially abundant bacterial species between FMT and Combo groups. **(D)** Heatmap showing the correlations of species significantly different between FMT and Combo groups. **(E)** Abundance of specific species in different groups. Data are represented as mean ± SD. *, *p*-value < 0.05; **, *p*-value < 0.01; ***, *p*-value < 0.001.

To investigate whether FMT improved the effects of aPD-1 by refining the gut microbiome, we next performed metagenomic analysis to examine FMT-induced changes of gut microbial composition and gene function. The PCA plot showed an obvious group-based clustering pattern among groups with or without FMT treatment, indicating that FMT significantly changed the composition of gut microbiota (Adonis R^2 =^ 0.58, *p*-value=0.000167), while the change caused by aPD-1 was less remarkable (**Figure 2A**). FMT were associated with, at the family level, the decrease of the relative abundance of *Bifidobacteriaceae*, *Porphyromonadaceae*, *Verrucomicrobiaceae*, and the increase of *Desulfovibrionaceae* and *Bacteroidaceae* (**Figure 2B**).

Nineteen significantly differential abundant species between the Combo group and aPD-1 group were identified using linear discriminant analysis. The relative abundance of multiple *Bacteroides* species (*B. thetaiotaomicron, B. stercoris, B. salyersiae, B. fragilis, B. cellulosilyticus, B. uniformis*, and *B. massiliensis*) and *Parabacteroides* species (*P. distasonis* and *P.* unclassified) were significantly increased in the mice treated with the combination of FMT and aPD-1, compared to those treated with aPD-1 alone. We also observed the decreased abundance of the abundance of *Clostridium* sp HGF2*, Enterococcus hirae, Dorea* 52*, Lactobacillus murinus*, and *Bacteroides ovatus* were observed (**Figures 2C**, **E**, **S1A**, **B**). In addition, we observed the abundance of specific bacteria, including *Alistipes indistinctus, Faecalibacterium prausnitzii, Bacteroides vulgatus*, and *Oscillibacter* unclassified were enriched, while *Bifidobacterium pseudolongum* were decreased by FMT treatment(*p*<0.05), and opposite trends were observed in aPD-1 group (**Figure S1B**).

The abundance of the aforementioned *Bacteroides* species showed a strong positive correlation with each other (|coefficient value|>0.6, *p*<0.05), as well as a negative correlation with *Enterococcus hirae, Dorea* 52, and *Lactobacillus murinus* (**Figure 2D**). Interestingly, the abundance of *Bacteroides ovatus* correlated negatively with the abundance of most of the FMT-upregulated species (**Figure 2D**). In a nutshell, our results showed that FMT altered the composition of gut microbiota, particularly *Bacteroides* (the increased *B. thetaiotaomicron, B. fragilis*, and *B. cellulosilyticus* and the decreased *B. ovatus*).

### FMT upregulated microbial biosynthetic pathways of nucleotides and amino acids

**Figure 3:**
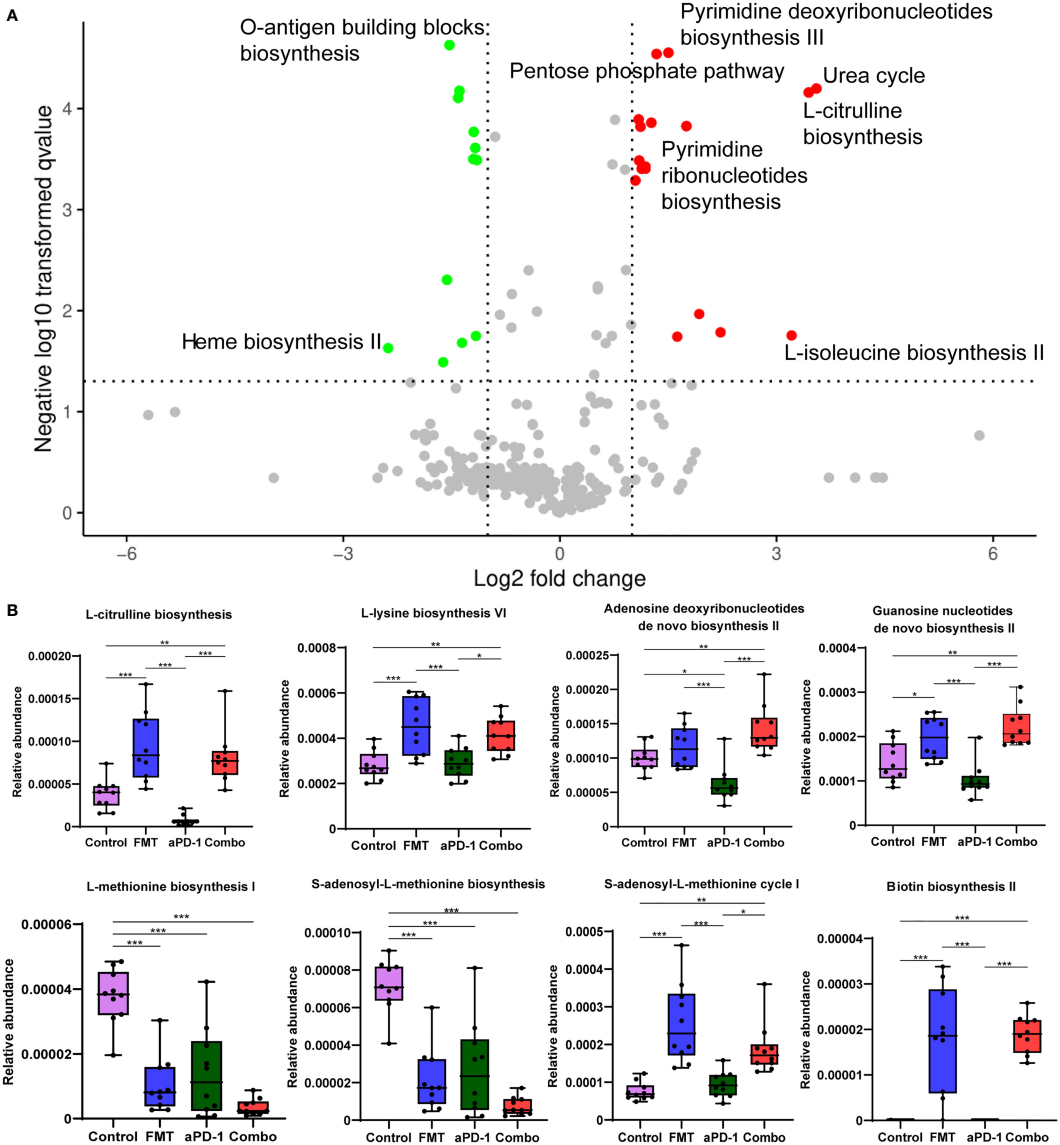
The effect of FMT and PD-1 antibody administration on gut metagenomic gene pathways. **(A)** Volcano plot showing differentially expressed microbial gene pathways between Combo and aPD-1 groups. **(B)** Abundance of specific gene pathways in different groups. Data are represented as mean ± SD. *, *p*-value < 0.05; **, *p*-value < 0.01; ***, *p*-value < 0.001.

Other than microbial composition, we also examined microbial gene functional changes upon treatments, which may influence gastrointestinal and systemic physiology. Compared to those of aPD-1 group, 27 differently abundant pathways out of 491 were identified in the combination group (|log2FC|>1, *p*-adjusted<0.05), indicating the potential microbial contribution towards better anti-PD-1 efficacy induced by FMT (**Figure 3A**). We observed that the anabolic pathways of several amino acids, including ornithine, histidine, lysine, citrulline, and isoleucine were significantly enriched by FMT treatment. And the pathways of nucleotides *de novo* biosynthesis, including pyrimidine deoxyribonucleotides, guanosine nucleotides, and adenosine nucleotides were significantly up-regulated in FMT and Combo group. Notably, the pathways of methionine and S-adenosyl-L-methionine (SAM) biosynthesis were significantly decreased, and pathways of S-adenosyl-L-methionine cycle I was increased by FMT treatment. Moreover, the pathways of coenzyme A biosynthesis I, O-antigen building blocks biosynthesis, and heme biosynthesis II were enriched in the aPD-1 group, while down-regulated in the Combo group. Furthermore, the pathway of biotin biosynthesis was significantly up-regulated by FMT treatment (**Figure 3B**, **Figure S2**).

### FMT and aPD-1 synergistically remodeled mouse plasma metabolome

**Figure 4:**
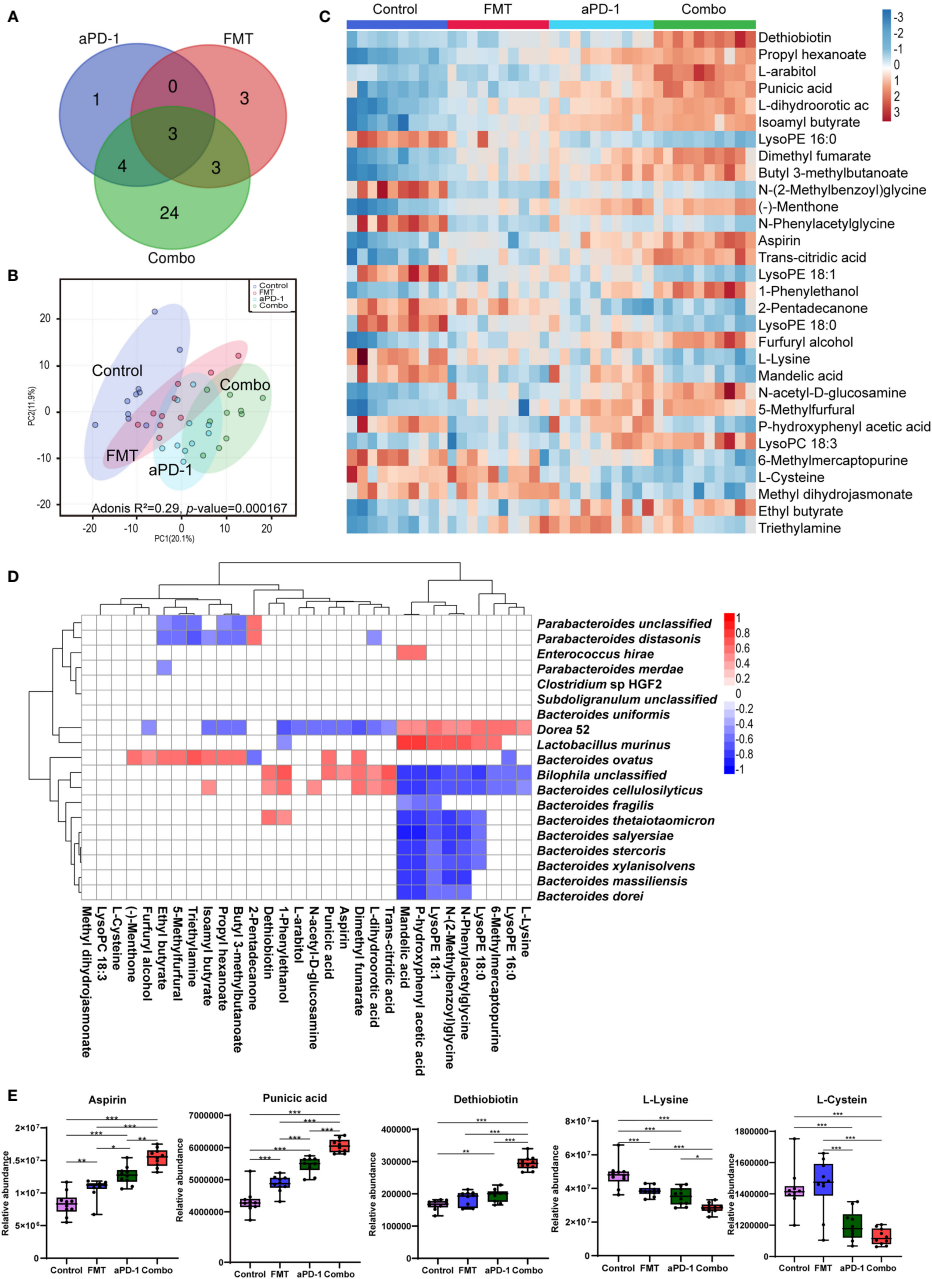
FMT altered plasma metabolites in CT-26 tumor-bearing mice receiving anti-PD-1 therapy. **(A)** Venn diagrams showing number of significantly changed metabolites in each group after treatment. **(B)** PCA plot of metabolomic results. **(C)** Heatmap of differentially abundant metabolites using one-way analysis of variance. **(D)** The correlations between metabolites and microorganism. **(E)** Abundance of specific metabolites in different groups. Data are represented as mean ± SD. *, *p*-value < 0.05; **, *p*-value < 0.01; ***, *p*-value < 0.001.

Metabolomic analyses were performed to examine the systemic change caused by FMT in tumor-bearing mice. Among a total number of 369 metabolites detected, the abundance of 8, 9, 34 metabolites were altered (*p*-adjusted < 0.05) following aPD-1, FMT, and Combo treatment, respectively, suggesting the synergistic effect of the combinational treatment (**Figure 4A**, **Table S1**). Abundance of 24 metabolites were altered upon the combinational treatment but not upon the treatment of FMT or aPD-1 alone, including the up-regulated kynurenic acid, estrone 3-sulfate and N -acetyl-D-glucosamine, and down-regulated glycine, nicotinamide and salicyluric acid (**Table S1**). The PCA plot also showed the distinct mouse plasma metabolome after different treatments (**Figure 4B**) (Adonis R^2^ = 0.29, *p*-value = 0.000167).

Top 30 most differentially abundant metabolites among the four groups were identified based on the FDR values from one-way ANOVA analysis (**Figure 4C**). Compared with the PD-1 group, dethiobiotin, punicic acid, aspirin, L-arabitol, N-acetyl-D-glucosamine, L-dihydroorotic acid, dimethyl fumarate, trans-citridic acid, 1-Phenylethanol were significantly increased in the Combo group (p<0.01). While lysoPE (16:0), triethylamine, glycine, L-lysine, mandelic acid, L-glutamic acid, L-phenylalanine were significantly decreased (p<0.01) (**Figures 4C**, **E**, **S3**). The results indicated that combinational treatment of FMT and aPD-1 significantly altered plasma metabolic profiles. Furthermore, amino acids, including N-(2-Methylbenzoyl) glycine, N-phenyl acetyl glycine, glycine, L-proline, L-cysteine, L-serine and L-lysine were significantly down-regulated in the Combo group (p<0.05). Notably, the abundance of dethiobiotin, propyl hexanoate, and N-acetyl-D-glucosamine were significantly up-regulated in the Combo group (**Figures 4C**, **E**, **S3**).

To better understand the involvement of specific bacteria species in the alteration of host metabolism, correlation between plasma metabolites and the abundance of specific bacteria species were investigated. High abundance of *Bacteroides* species, such as *B. thetaiotaomicron, B. stercoris, B. salyersiae, B. cellulosilyticus*, was positively correlated with the low abundance of lysoPE (18:0), lysoPE (18:1), N-phenyl acetyl glycine, N-(2-Methylbenzoyl) glycine in plasma, and opposite trends were observed in *B. ovatus* and *Lactobacillus murinus* (**Figure 4D**). This result suggests a potential link among commensal microorganisms, differentially abundant metabolites, and treatment outcomes of anti-PD-1 therapeutic efficacy.

## Discussion

Fecal microbiota transplantation from patients who responded to ICIs combined with ICIs exerts as a promising approach to treating melanoma (17). However, the detailed mechanisms and the applicability of this therapy are required to be further evaluated in multiple cancer types, such as colorectal cancer and lung cancer. Moreover, FMT using feces of cancer patients might carry safety risks such as detrimental pathogens or pathobionts; therefore, it’s necessary to examine the effect of FMT using feces from healthy donors. In this study, our multi-omics investigation shows the potential synergistic effects of FMT using feces from healthy screened donors and anti-PD-1 therapy, in the treatment of mice bearing colorectal tumor.

A wide range of commensal bacterial species have been reported to be associated with the enhanced efficacy of ICIs, including *B. thetaiotaomicron* (23), *B. fragilis* (24), *B. cellulosilyticus* (25), *Parabacteroides distasonis* (26)*, B. salyersiae* (27), and *B. uniformis* (13). In this study, our metagenomic analysis showed that FMT significantly upregulated the abundance of those potentially beneficial species, particularly those species from *Bacteroides* genus (**Figures 2C**, **E**). The reshaped microbiota caused by FMT might be associated with the refinement of tumor immune microenvironment (TIME) (28). Previous literature shows that *B. thetaiotaomicron*, which is most significantly upregulated by FMT in our data, has been reported to induce immune responses in dendritic cells (e.g. the expression of IL-10) and mediate intestinal homeostasis (29). *B. thetaiotaomicron* is also able to inhibit the growth of CRC cells *via* its metabolite propionate (23). Another *Bacteroides* species *B. fragilis* is associated with the favorable clinical outcome of CTLA-4 inhibitors (24) *via* inducing regulatory T cells to secrete IL-10 through the immunomodulatory molecule polysaccharide A (PSA) of *B. fragilis* (30). Additional immunomodulatory function of *B. fragilis* includes producing unique alpha-galactose ceramides (BfaGC) and subsequently activating NKT cells (e.g. upregulating IL-2 expression) (31). More recently, *B. cellulosilyticus* has been reported to be enriched in humanized microbiome mouse model of glioma and is a potential contributor to the enhanced efficacy of anti-PD-1 therapy (25). *B. cellulosilyticus* might modulate host immunity *via* its specific zwitterionic capsular polysaccharides (ZPSs) which can activate IL-10^+^ regulatory T cells to secrete IL-10 (25). Notably, the abundance of upregulated *Bacteroides* species showed a strong positive correlation with each other (**Figure 2D**), suggesting their potential symbiotic link. Furthermore, several bacterial species which showed an up-regulation in the Combo group, *Bilophila wadsworthia* and *Lachnospiraceae bacterium* have not been reported previously. Their roles in anti-PD-1 treatment would be very interesting to investigate.

The abundance of two potentially detrimental species, *B. ovatus* and *Lactobacillus murinus*, were significantly decreased by FMT (**Figures 2C**, **E**). It was previously reported that the abundance of *B. ovatus* was associated with shorter progression-free survival (PFS) in melanoma patients receiving immunotherapy (32). *B. ovatus* might affect host immunity *via* inducing IgA and other approaches (33). In addition, the outgrowth of *L. murinus* is considered to impair gut metabolic function and exacerbate intestinal dysbiosis (34), therefore the depletion of *L. murinus* led by FMT may attenuate the microbial dysbiosis. Our metagenomic results are in line with the previously published studies that FMT could reshape the composition of both beneficial and harmful bacteria in the gut microbiome upon the anti-PD-1 treatment, which might result in the enhanced therapeutic efficacy.

Microbial gene functions and host metabolome were also reshaped by FMT in this study, which might benefit the efficacy of immunotherapy. Microbial gene pathways including nucleotides and amino acid biosynthesis pathways (e.g., pyrimidine deoxyribonucleotides, guanosine nucleotides, ornithine, isoleucine) were enriched after FMT, whereas methionine and SAM biosynthesis pathways were significantly downregulated (**Figure 3A, B**). Methionine is involved in the pathogenesis of cancer (35), and negatively related to the efficacy of radiotherapy (36). SAM, a universal methyl donor, is formed from methionine and has been reported to be associated with metastasis and recurrence in colorectal cancer patients (37). Inhibition of the production of methionine and SAM might contribute to the tumor regression. Furthermore, our metabolomics analysis showed higher abundance of aspirin which can inhibit the growth of *Fusobacterium nucleatum* (a detrimental bacteria species which aggravates colorectal cancer) after FMT treatment (38). Likewise, punicic acid was regulated upon FMT. The potent anti-tumor effect of punicic acid might play a role in tumor control (39, 40). Lastly, the abundance of several amino acids was also decreased in the plasma, including glycine, serine, and cysteine (**Figure 4C**, **E**, **Figure S3**). Previous research reported that the growth and proliferation of cancer cells require serine and glycine, and limiting exogenous serine and glycine could inhibit tumor growth in mouse models of colon cancer (41, 42). Moreover, the combinational treatment up-regulated the abundance of blood metabolite kynurenic acid, which has been reported to inhibit proliferation of colon cancer and renal cancer cells (43). To summarize, the enhanced efficacy of anti-PD-1 therapy led by FMT might be mediated by the altered microbial genome and blood metabolome.

The limitations of this study include the lack of experimental validation of the aforementioned bacterial species, metabolic pathways and changes of immune cells. Also, the synergistic effect exerted in mouse model may vary from that in the clinic. Further clinical investigation is being conducted in our laboratory and is anticipated to shed light on the detailed mechanisms of the promising combined use of FMT and anti-PD-1 therapy.

## Conclusion

In summary, our study provides novel insight into the synergetic effects of microbiota transplantation and anti-PD-1 therapy in treating colorectal cancer, including the remodeling of gut microbiota and plasma metabolome. Our results suggest that *Bacteroides*, including the FMT-increased *B. thetaiotaomicron, B. fragilis*, and *B. cellulosilyticus* and decreased *B. ovatus* might contribute to the improved the efficacy of anti-PD-1 therapy. This work provides a potential mechanistic basis to further understand the role of FMT combined with anti-PD-1 therapy in treating various cancer types including colorectal cancer.

## Data availability statement

The original contributions presented in the study are publicly available. This data can be found here: https://www.ncbi.nlm.nih.gov/bioproject/PRJNA799796.

## Ethics statement

The animal study was reviewed and approved by Crown Biosciences Co. Ltd. (Taicang, China).

## Author contributions

HH, YT, YY and WZ conceived the study. JH, XZ, WK and HH conducted the experiments. JH, XZ, HH, WK, YM and HZ performed data analysis and interpretation. YC, YH, YT, WZ and YY supervised and financially supported the study. JH, XZ, WK, WZ and YY wrote the manuscript with extensive input from all authors. All authors contributed to the article and approved the submitted version.

## Funding

This study received funding from National Key Research and Development Program of China (2020YFA0907800) and Shenzhen Science and Technology Innovation Program (KQTD20200820145822023). The funders were not involved in the study design, collection, analysis, interpretation of data, the writing of this article or the decision to submit it for publication.

## Conflict of interest

Authors XZ, HH, YT and YY are employed by Xbiome Biotech Co. Ltd.

The remaining authors declare that the research was conducted in the absence of any commercial or financial relationships that could be construed as a potential conflict of interest.

## Publisher’s note

All claims expressed in this article are solely those of the authors and do not necessarily represent those of their affiliated organizations, or those of the publisher, the editors and the reviewers. Any product that may be evaluated in this article, or claim that may be made by its manufacturer, is not guaranteed or endorsed by the publisher.

## Supplementary material

Supplementary Table 1 Differentially abundant blood metabolites of the CT26 tumor-bearing mice upon different types of treatment. *p*-adjusted value < 0.05 is considered as statistically significant.

## References

[1] Schoenfeld AJ Hellmann MD. Acquired resistance to immune checkpoint inhibitors. Cancer Cell (2020) 37(4):443–55. doi: 10.1016/j.ccell.2020.03.017 PMC7182070 32289269

[2] Alsaab HO Sau S Alzhrani R Tatiparti K Bhise K Kashaw SK. PD-1 and PD-L1 checkpoint signaling inhibition for cancer immunotherapy: mechanism, combinations, and clinical outcome. Front Pharmacol (2017) 8:561. doi: 10.3389/fphar.2017.00561 28878676 PMC5572324

[3] Zhang Y Chen L. Classification of advanced human cancers based on tumor immunity in the microenvironment (time) for cancer immunotherapy. JAMA Oncol (2016) 2(11):1403–4. doi: 10.1001/jamaoncol.2016.2450 PMC5492376 27490017

[4] Kim TK Herbst RS Chen L. Defining and understanding adaptive resistance in cancer immunotherapy. Trends Immunol (2018) 39(8):624–31. doi: 10.1016/j.it.2018.05.001 PMC6066429 29802087

[5] Puccini A Lenz HJ. Colorectal cancer in 2017: Practice-changing updates in the adjuvant and metastatic setting. Nat Rev Clin Oncol (2018) 15(2):77–8. doi: 10.1038/nrclinonc.2017.185 PMC7491976 29182161

[6] Ganesh K Stadler ZK Cercek A Mendelsohn RB Shia J Segal NH. Immunotherapy in colorectal cancer: rationale, challenges and potential. Nat Rev Gastroenterol Hepatol (2019) 16(6):361–75. doi: 10.1038/s41575-019-0126-x PMC7295073 30886395

[7] Cox LM Yamanishi S Sohn J Alekseyenko AV Leung JM Cho I. Altering the intestinal microbiota during a critical developmental window has lasting metabolic consequences. Cell (2014) 158(4):705–21. doi: 10.1016/j.cell.2014.05.052 PMC4134513 25126780

[8] Gao Y Bi D Xie R Li M Guo J Liu H. *Fusobacterium nucleatum* enhances the efficacy of pd-l1 blockade in colorectal cancer. Signal Transduct Targeted Ther (2021) 6(1):398. doi: 10.1038/s41392-021-00795-x PMC8602417 34795206

[9] Huang Y Zhu N Zheng X Liu Y Lu H Yin X. Intratumor microbiome analysis identifies positive association between *megasphaera* and survival of chinese patients with pancreatic ductal adenocarcinomas. Front Immunol (2022) 13:785422. doi: 10.3389/fimmu.2022.785422 35145519 PMC8821101

[10] Routy B Le Chatelier E Derosa L Duong CPM Alou MT Daillère R. Gut microbiome influences efficacy of pd-1-based immunotherapy against epithelial tumors. Sci (New York NY) (2018) 359(6371):91–7. doi: 10.1126/science.aan3706 29097494

[11] Gopalakrishnan V Spencer CN Nezi L Reuben A Andrews MC Karpinets TV. Gut microbiome modulates response to anti-pd-1 immunotherapy in melanoma patients. Sci (New York NY) (2018) 359(6371):97–103. doi: 10.1126/science.aan4236 PMC5827966 29097493

[12] Matson V Fessler J Bao R Chongsuwat T Zha Y Alegre ML. The commensal microbiome is associated with anti-pd-1 efficacy in metastatic melanoma patients. Sci (New York NY) (2018) 359(6371):104–8. doi: 10.1126/science.aao3290 PMC6707353 29302014

[13] Si W Liang H Bugno J Xu Q Ding X Yang K. *Lactobacillus rhamnosus* GG induces cGAS/STING-dependent type I interferon and improves response to immune checkpoint blockade. Gut (2021) 71:521–533. doi: 10.1136/gutjnl-2020-323426 PMC8710942 33685966

[14] Toker J Arora R Wargo JA. The microbiome in immuno-oncology. Adv Exp Med Biol (2020) 1244:325–34. doi: 10.1007/978-3-030-41008-7_19 32301026

[15] Borody TJ Eslick GD Clancy RL. Fecal microbiota transplantation as a new therapy: from *Clostridioides difficile* infection to inflammatory bowel disease, irritable bowel syndrome, and colon cancer. Curr Opin Pharmacol (2019) 49:43–51. doi: 10.1016/j.coph.2019.04.017 31173991

[16] Baruch EN Youngster I Ben-Betzalel G Ortenberg R Lahat A Katz L. Fecal microbiota transplant promotes response in immunotherapy-refractory melanoma patients. Sci (New York NY) (2021) 371(6529):602–9. doi: 10.1126/science.abb5920 33303685

[17] Davar D Dzutsev AK McCulloch JA Rodrigues RR Chauvin JM Morrison RM. Fecal microbiota transplant overcomes resistance to anti-pd-1 therapy in melanoma patients. Sci (New York NY) (2021) 371(6529):595–602. doi: 10.1126/science.abf3363 PMC8097968 33542131

[18] Huang J Liu D Wang Y Liu L Li J Yuan J. Ginseng polysaccharides alter the gut microbiota and kynurenine/tryptophan ratio, potentiating the antitumour effect of antiprogrammed cell death 1/programmed cell death ligand 1 (anti-PD-1/PD-L1) immunotherapy. Gut (2021), 71:1–12. doi: 10.1136/gutjnl-2020-321031 34006584 PMC8921579

[19] Wang Y Tang J Lv Q Tan Y Dong X Liu H. Establishment and resilience of transplanted gut microbiota in aged mice. iScience (2022) 25(1):103654. doi: 10.1016/j.isci.2021.103654 35024588 PMC8733228

[20] Beghini F McIver LJ Blanco-Míguez A Dubois L Asnicar F Maharjan S. Integrating taxonomic, functional, and strain-level profiling of diverse microbial communities with biobakery 3. eLife (2021) 10:e65088. doi: 10.7554/eLife.65088 33944776 PMC8096432

[21] Chen W Gong L Guo Z Wang W Zhang H Liu X. A novel integrated method for large-scale detection, identification, and quantification of widely targeted metabolites: application in the study of rice metabolomics. Mol Plant (2013) 6(6):1769–80. doi: 10.1093/mp/sst080 23702596

[22] Chong J Wishart DS Xia J. Using metaboanalyst 4.0 for comprehensive and integrative metabolomics data analysis. Curr Protoc Bioinf (2019) 68(1):e86. doi: 10.1002/cpbi.86 31756036

[23] Ryu TY Kim K Han TS Lee MO Lee J Choi J. Human gut-microbiome-derived propionate coordinates proteasomal degradation *via* hectd2 upregulation to target EHMT2 in colorectal cancer. ISME J (2022), 16:1–17. doi: 10.1038/s41396-021-01119-1 34972816 PMC9038766

[24] Vétizou M Pitt JM Daillère R Lepage P Waldschmitt N Flament C. Anticancer immunotherapy by CTLA-4 blockade relies on the gut microbiota. Sci (New York NY) (2015) 350(6264):1079–84. doi: 10.1126/science.aad1329 PMC4721659 26541610

[25] Neff CP Rhodes ME Arnolds KL Collins CB Donnelly J Nusbacher N. Diverse intestinal bacteria contain putative zwitterionic capsular polysaccharides with anti-inflammatory properties. Cell Host Microbe (2016) 20(4):535–47. doi: 10.1016/j.chom.2016.09.002 PMC5113727 27693306

[26] Gao G Ma T Zhang T Jin H Li Y Kwok LY. Adjunctive probiotic *Lactobacillus rhamnosus* probio-M9 administration enhances the effect of anti-pd-1 antitumor therapy *via* restoring antibiotic-disrupted gut microbiota. Front Immunol (2021) 12:772532. doi: 10.3389/fimmu.2021.772532 34970262 PMC8712698

[27] Derosa L Routy B Fidelle M Iebba V Alla L Pasolli E. Gut bacteria composition drives primary resistance to cancer immunotherapy in renal cell carcinoma patients. Eur Urol (2020) 78(2):195–206. doi: 10.1016/j.eururo.2020.04.044 32376136

[28] Wong-Rolle A Wei HK Zhao C Jin C. Unexpected guests in the tumor microenvironment: microbiome in cancer. Protein Cell (2021) 12(5):426–35. doi: 10.1007/s13238-020-00813-8 PMC8106554 33296049

[29] Durant L Stentz R Noble A Brooks J Gicheva N Reddi D. *Bacteroides thetaiotaomicron*-derived outer membrane vesicles promote regulatory dendritic cell responses in health but not in inflammatory bowel disease. Microbiome (2020) 8(1):88. doi: 10.1186/s40168-020-00868-z 32513301 PMC7282036

[30] Dasgupta S Erturk-Hasdemir D Ochoa-Reparaz J Reinecker HC Kasper DL. Plasmacytoid dendritic cells mediate anti-inflammatory responses to a gut commensal molecule *via* both innate and adaptive mechanisms. Cell Host Microbe (2014) 15(4):413–23. doi: 10.1016/j.chom.2014.03.006 PMC4020153 24721570

[31] Oh SF Praveena T Song H Yoo JS Jung DJ Erturk-Hasdemir D. Host immunomodulatory lipids created by symbionts from dietary amino acids. Nature (2021) 600(7888):302–7. doi: 10.1038/s41586-021-04083-0 PMC8999822 34759313

[32] Peters BA Wilson M Moran U Pavlick A Izsak A Wechter T. Relating the gut metagenome and metatranscriptome to immunotherapy responses in melanoma patients. Genome Med (2019) 11(1):61. doi: 10.1186/s13073-019-0672-4 31597568 PMC6785875

[33] Yang C Mogno I Contijoch EJ Borgerding JN Aggarwala V Li Z. Fecal IgA levels are determined by strain-level differences in bacteroides ovatus and are modifiable by gut microbiota manipulation. Cell Host Microbe (2020) 27(3):467–75.e6. doi: 10.1016/j.chom.2020.01.016 32075742 PMC7213796

[34] Hayashi A Mikami Y Miyamoto K Kamada N Sato T Mizuno S. Intestinal dysbiosis and biotin deprivation induce alopecia through overgrowth of *Lactobacillus murinus* in mice. Cell Rep (2017) 20(7):1513–24. doi: 10.1016/j.celrep.2017.07.057 28813664

[35] Sanderson SM Gao X Dai Z Locasale JW. Methionine metabolism in health and cancer: a nexus of diet and precision medicine. Nat Rev Cancer (2019) 19(11):625–37. doi: 10.1038/s41568-019-0187-8 31515518

[36] Gao X Sanderson SM Dai Z Reid MA Cooper DE Lu M. Dietary methionine influences therapy in mouse cancer models and alters human metabolism. Nature (2019) 572(7769):397–401. doi: 10.1038/s41586-019-1437-3 31367041 PMC6951023

[37] Zhang Y Yu H Zhang J Gao H Wang S Li S. Cul4A-DDB1-mediated monoubiquitination of phosphoglycerate dehydrogenase promotes colorectal cancer metastasis *via* increased s-adenosylmethionine. J Clin Invest (2021) 131(21):1–18. doi: 10.1172/jci146187 PMC8553555 34720086

[38] Brennan CA Nakatsu G Gallini Comeau CA Drew DA Glickman JN Schoen RE. Aspirin modulation of the colorectal cancer-associated microbe *Fusobacterium nucleatum* . mBio (2021) 12(2):1–16. doi: 10.1128/mBio.00547-21 PMC8092249 33824205

[39] Mete M Ünsal Ü Aydemir I Sönmez PK Tuglu MI. Punicic acid inhibits glioblastoma migration and proliferation v*ia* the PI3k/AKT1/mTOR signaling pathway. Anti-Cancer Agents Medicinal Chem (2019) 19(9):1120–31. doi: 10.2174/1871520619666190405112507 30950355

[40] Yuan G Tan M Chen X. Punicic acid ameliorates obesity and liver steatosis by regulating gut microbiota composition in mice. Food Funct (2021) 12(17):7897–908. doi: 10.1039/d1fo01152a 34241611

[41] Maddocks ODK Athineos D Cheung EC Lee P Zhang T van den Broek NJF. Modulating the therapeutic response of tumours to dietary serine and glycine starvation. Nature (2017) 544(7650):372–6. doi: 10.1038/nature22056 28425994

[42] Muthusamy T Cordes T Handzlik MK You L Lim EW Gengatharan J. Serine restriction alters sphingolipid diversity to constrain tumour growth. Nature (2020) 586(7831):790–5. doi: 10.1038/s41586-020-2609-x PMC7606299 32788725

[43] Walczak K Turski WA Rajtar G. Kynurenic acid inhibits colon cancer proliferation in vitro: Effects on signaling pathways. Amino Acids (2020) 46(10):2393–401. doi: 10.1007/s00726-014-1790-3 PMC4168223 25012123

