# Supplementary material for: Metagenomic and metabolomic analyses reveal synergistic effects of fecal microbiota transplantation and anti-PD-1 therapy on treating colorectal cancer
Source: Front Immunol. 2022 Jul 15;13:874922. doi: 10.3389/fimmu.2022.874922 (PMC9336524; doi:10.3389/fimmu.2022.874922)
Supplement: Supplementary Table 1 — Differentially abundant blood metabolites of the CT26 tumor-bearing mice upon different types of treatment. p-adjusted value < 0.05 is considered as statistically significant. [file Table_1.docx]

**Table S1.** Differentially abundant blood metabolites of the CT26 tumor-bearing mice upon different types of treatment. *p*-adjusted value < 0.05 is considered as statistically significant.

| **Treatment** | **Number of metabolites** | **Up-regulated metabolite** | **Down-regulated metabolite** |
| --- | --- | --- | --- |
| Combo, FMT, aPD-1 | 3 | \|  \|  \| \| --- \| --- \| \|  \|  \| \|  \|  \| | N-(2-methylbenzoyl)glycine  N-phenylacetylglycine  Phenylacetyl-L-glutamine |
| Combo, aPD-1 | 4 | \| δ-valerolactam \| \| --- \| \| Sn-Glycero-3-phosphocholine \| \| Propyl hexanoate \| | 2,4-di-tert-butylphenol |
| Combo, FMT | 3 | \|  \| \| --- \| \|  \| \|  \| | P–hydroxyphenylacetic acid  Hyodeoxycholic acid  Mandelic acid |
| aPD-1 | 1 | N-acetyl-L-valine |  |
| FMT | 3 | \| Hippuric acid \| \| --- \| \| Dihydroactinidiolide \| | Chenodeoxycholic acid |
| Combo | 24 | \| Kynurenic acid \| \| --- \| \| Estrone 3-sulfate \| \| N-acetyl-D-glucosamine \| | 12-HETE  3-Hydroxyhippuric acid  Indolelactic acid  Glycine  Salicyluric acid  Isonicotinic acid  3-Indolepropionic acid  9-HpODE  L-3-Phenyllactic acid  LysoPE 18:0  Hydroxyphenyllactic acid9,10-Dihydroxy-12Z-Octadecenoic acid  Nicotinamide  3-tert-Octylphenol  LysoPE 16:0  L-Erythrulose  m-Coumaric acid  Formononetin  (±)12-hydroxy-5Z,8Z,10E,14Z-eicosatetraenoic acid  LysoPE 18:1  Phenyllactate |
