# Supplementary material for: Metagenomic and metabolomic analyses reveal synergistic effects of fecal microbiota transplantation and anti-PD-1 therapy on treating colorectal cancer
Source: Front Immunol. 2022 Jul 15;13:874922. doi: 10.3389/fimmu.2022.874922 (PMC9336524; doi:10.3389/fimmu.2022.874922)
Supplement: Supplementary file 2 [file Image_1.pdf]

Supplementary figures

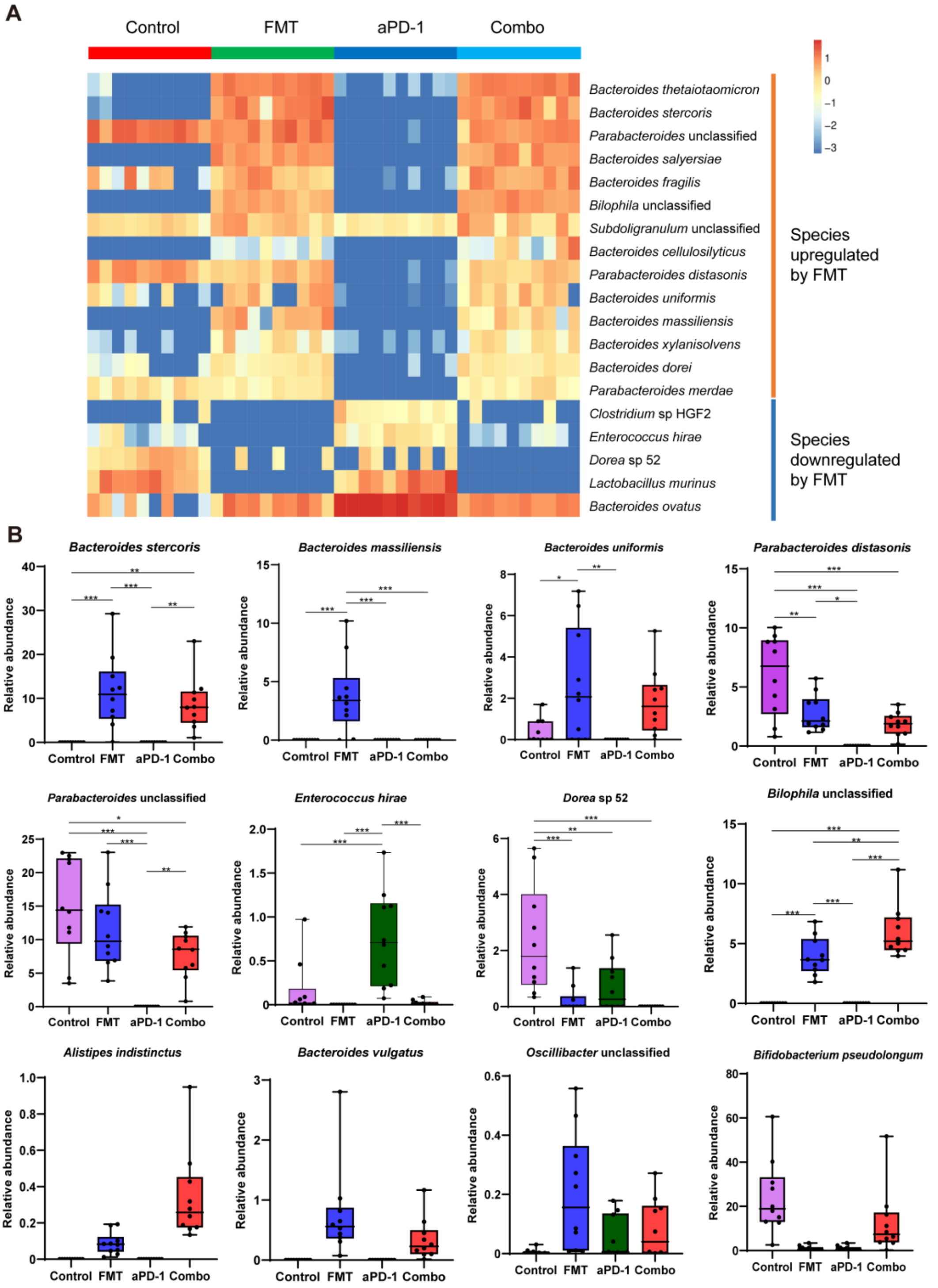

Figure S1. FMT altered the composition of gut microbiota in CT-26 tumor-bearing mice receiving anti-PD-1 therapy. (A) Heatmap of differentially abundant bacterial species. (B) Abundance of specific bacterial species in different groups. Data are represented as mean ± SD. *, p<0.05; **, p<0.01; ***, p<0.001.

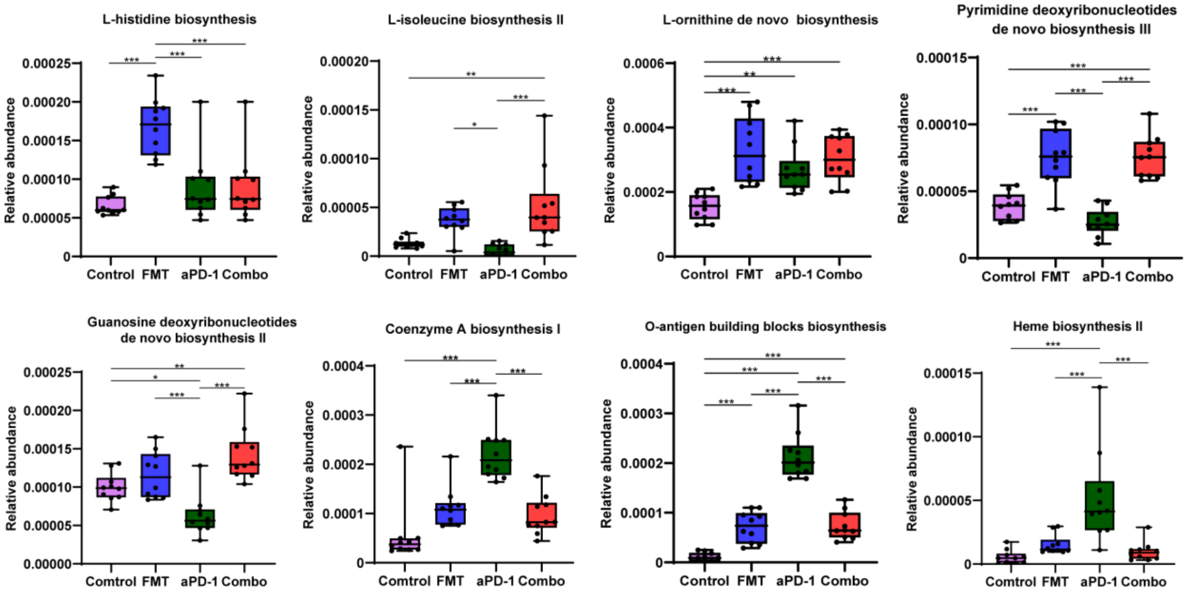

Figure S2. Abundance of significant gene pathways following different treatments. *, $p<0.05$; **, $p<0.01$; ***, $p<0.001$.

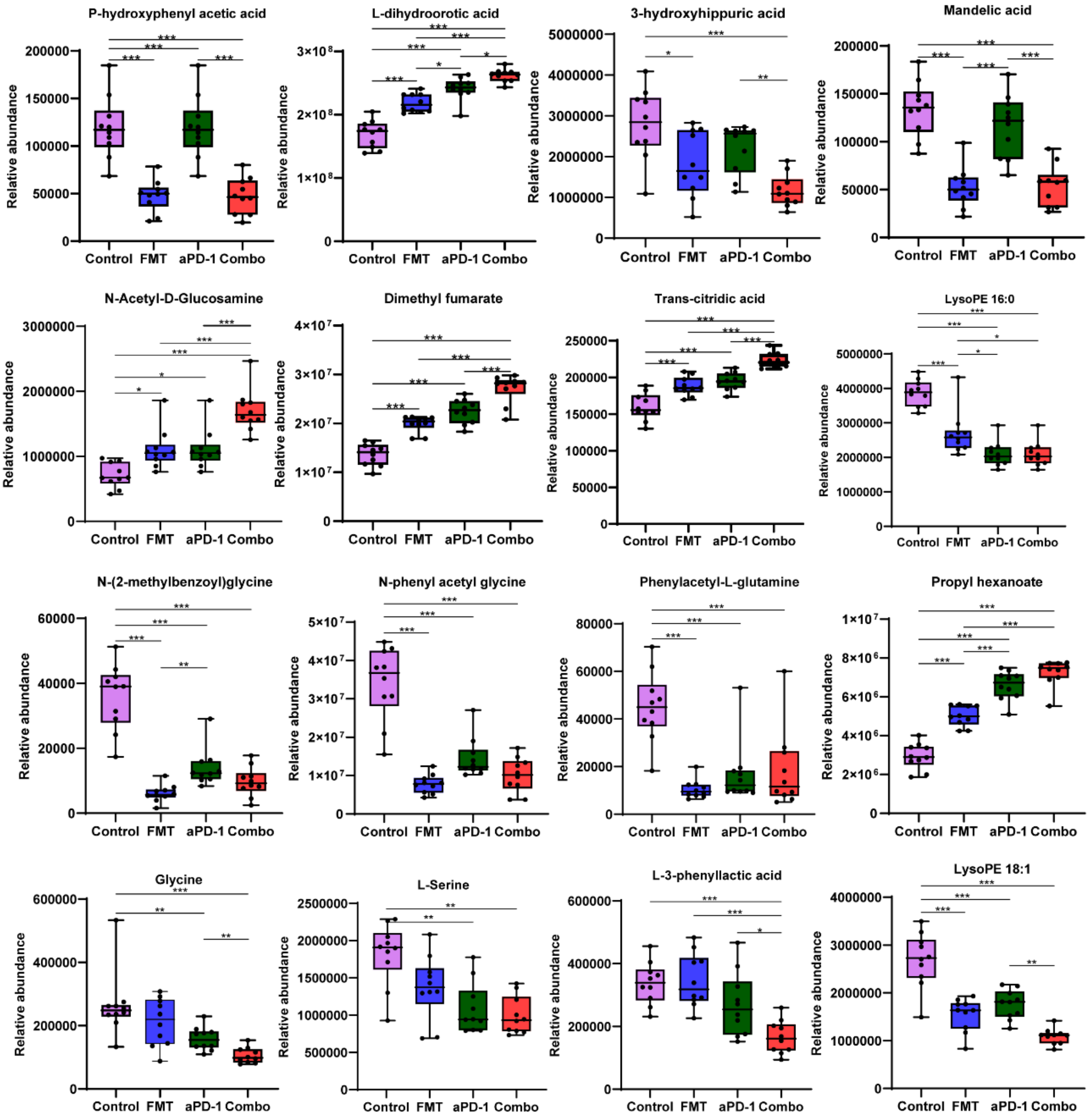

Figure S3. Abundance of significant metabolites following different treatments. Data are represented as mean ± SD. *, p<0.05; **, p<0.01; ***, p<0.001.
